# Effect of early intravenous magnesium sulfate on clinical outcomes in patients with moderate to severe traumatic brain injury: a study protocol for a randomized controlled trial

**DOI:** 10.3389/fmed.2026.1900332

**Published:** 2026-09-15

**Authors:** Hong Xi, Yifan Liu, Lixiong Xue, Qiang Li, Ying Zhang, Hailong Zhang, Junyan Zhang, Zheng Guo

**Affiliations:** 1 Department of Anesthesiology, Third Hospital of Shanxi Medical University, Shanxi Bethune Hospital, Shanxi Academy of Medical Sciences, Tongji Shanxi Hospital, Taiyuan, China; 2 Department of Neurosurgery, Shanxi Bethune Hospital, Shanxi Academy of Medical Sciences, Tongji Shanxi Hospital, Third Hospital of Shanxi Medical University, Taiyuan, China; 3 Department of Laboratory Medicine, Shanxi Bethune Hospital, Shanxi Academy of Medical Sciences, Tongji Shanxi Hospital, Third Hospital of Shanxi Medical University, Taiyuan, China; 4 Department of Epidemiology and Evidence-Based Medicine, Shanxi Bethune Hospital, Shanxi Academy of Medical Sciences, Tongji Shanxi Hospital, Third Hospital of Shanxi Medical University, Taiyuan, China; 5 College of Anesthesia, Shanxi Medical University, Taiyuan, China; 6 Department of Anesthesia, Second Hospital of Shanxi Medical University, Taiyuan, China; 7 Key Laboratory of Cellular Physiology, Shanxi Medical University, Taiyuan, China

**Keywords:** acute respiratory distress syndrome, brain-lung axis, magnesium sulfate, neuroinflammation, traumatic brain injury

## Abstract

**Background:**

Traumatic brain injury (TBI) is associated with substantial mortality and long-term disability worldwide. Acute respiratory distress syndrome (ARDS), as a common complication in moderate-to-severe TBI, further aggravates clinical outcomes and resource utilization. However, effective interventions to prevent TBI-associated ARDS remain limited. This trial aims to evaluate whether early intravenous magnesium sulfate reduces the risk of ARDS and improves clinical outcomes in patients with moderate-to-severe TBI.

**Methods:**

This protocol outlines a randomized, controlled, double-blind clinical trial involving 164 adults (18–75 years) with moderate-to-severe TBI (Glasgow Coma Scale [GCS] 3–12) within 8 h of injury. Participants will be stratified by baseline GCS (3–8 vs. 9–12) and randomized 1:1 to the intravenous magnesium sulfate group (initial 2 g over 30 min within 8 h, then 2 g daily for 5 days; total 12 g) or an identical placebo group. Both groups will receive guideline-directed standard TBI care. The primary endpoint is a 28-day hierarchical composite (all-cause mortality, then new-onset ARDS within days 1–7, then ≤ 20% increase in PaO₂/FiO₂ AUC from baseline over days 0–5). The hierarchical composite primary endpoint will be analyzed using the win-ratio method. Secondary outcomes include ICU and hospital length of stay, complications, and serial biomarker changes. Follow-up assessments will be conducted on day 28 and at 6 months post-treatment.

**Discussion:**

If effective, this low-cost intervention could be readily incorporated into standard TBI care and help prevent TBI-associated ARDS.

**Clinical Trial Registration:**

Chinese Clinical Trial Registry, identifier ChiCTR2600120684.

## Introduction

Traumatic brain injury (TBI) is a leading cause of death and disability worldwide, particularly among individuals aged 15–45 years (1–3). Moderate-to-severe TBI (Glasgow Coma Scale [GCS] 3–12) accounts for a substantial proportion of the global TBI burden (2). In 2021, approximately 20.8 million new TBI cases occurred globally (4). Beyond the initial mechanical insult, secondary injury cascades play a critical role in determining clinical outcomes (5). Up to 30% of patients with moderate-to-severe TBI develop acute respiratory distress syndrome (ARDS) within hours to days after injury, reflecting a harmful brain-lung interaction that significantly increases 28-day mortality, prolongs intensive care unit (ICU) stay, and worsens long-term functional outcomes (6–8). Management of these patients is particularly challenging, as lung-protective ventilation strategies may conflict with neuroprotective objectives, including maintaining adequate cerebral perfusion pressure, preventing hypercapnia, and controlling intracranial pressure (9). Current treatment for TBI-associated ARDS remains largely supportive, and no targeted pharmacological strategy has been established to prevent this complication. Following severe brain injury, sympathetic nervous system activation, catecholamine surge, systemic inflammation, oxidative stress, disruption of endothelial and epithelial barriers, and immune dysregulation collectively contribute to acute lung injury (10). Therefore, early interventions capable of modulating inflammation, oxidative stress, calcium homeostasis, and endothelial injury may have potential value in reducing pulmonary deterioration after TBI (11).

Magnesium status represents a modifiable biological factor in this context (12), as experimental and clinical evidence demonstrates that magnesium depletion exacerbates calcium-dependent excitotoxicity, oxidative stress, mitochondrial dysfunction, endothelial damage, and inflammatory activation (13, 14). These magnesium-sensitive processes are also implicated in pulmonary endothelial and epithelial injury, suggesting that magnesium homeostasis may represent a potential link between secondary brain injury and TBI-associated lung dysfunction (15, 16). Magnesium sulfate may confer protection by regulating ion homeostasis and dampening inflammation via several pathways, notably the HMGB1/TLR4/NF-*κ*B axis and TRPM7 kinase-related signaling (17–19).

However, most existing therapeutic strategies for TBI have focused almost exclusively on neuroprotection, with the brain-lung axis remaining a largely overlooked target. Despite the mechanistic rationale supporting its use, clinical evidence for magnesium sulfate in TBI remains inconclusive, largely due to an emphasis on neurological outcomes rather than pulmonary complications or the brain-lung axis. The landmark trial by Temkin et al. (20) reported no neuroprotective benefit and raised safety concerns, potentially related to the high serum magnesium targets (1.0–1.85 mmol/L) employed in that study. In contrast, the MOST trial uses a lower fixed-dose magnesium regimen with a target serum magnesium concentration of 1.0–1.5 mmol/L and extends the therapeutic focus from neuroprotection to TBI-associated pulmonary injury, using a lung-focused hierarchical composite endpoint together with serial mechanistic biomarker profiling. More recent smaller studies investigating magnesium sulfate in cases of mild TBI or severe diffuse axonal injury have yielded mixed results, with some suggesting short-term physiological or neurological improvements, yet none demonstrating consistent reductions in mortality or sustained functional recovery (21, 22). Similarly, certain perioperative neurosurgical studies of magnesium administration have indicated potential benefits in selected imaging or cognitive outcomes (23, 24); however, these findings cannot be directly extrapolated to patients with moderate-to-severe TBI or to pulmonary complications. Although clinical trials have evaluated the preventive and therapeutic effects of magnesium sulfate on pulmonary diseases such as ARDS (25), asthma (26) and chronic obstructive pulmonary disease (27), whether early use of magnesium sulfate can alleviate the pulmonary deterioration associated with traumatic brain injury remains an unresolved but clinically significant issue.

Given the emerging understanding of the brain-lung axis and the potential benefits of lower, fixed-dose magnesium regimens, we designed the Magnesium sulfate for Outcomes in Moderate to Severe Traumatic brain injury (MOST) trial to address this critical gap. This is the first dedicated investigation to evaluate whether early administration of magnesium sulfate reduces the incidence of ARDS and improves clinical outcomes in patients with moderate-to-severe TBI, thereby targeting the previously overlooked brain-lung axis. We hypothesize that early magnesium sulfate will reduce the risk of ARDS and improve clinical outcomes compared with placebo.

## Methods

### Study design

MOST is a single-center, randomized, double-blind, placebo-controlled phase II trial which will be implemented at Shanxi Bethune Hospital, a tertiary Grade-A academic medical center in Taiyuan, China. This protocol is reported in accordance with the SPIRIT 2025 statement: updated guideline for protocols of randomized trials (28). All study procedures will be performed according to standardized institutional protocols by physicians and nurses who have completed Good Clinical Practice (GCP) training. The schedule of enrollment, interventions, and outcome assessments is summarized in Table 1. The overall study design and participant flow are presented in Figure 1, and the detailed study timeline and follow-up schedule are illustrated in Figure 2.

**Table 1:** Participant timeline: schedule of enrollment, interventions, and assessments.

ENROLLMENT	Screening period	Core phase										Extension phase
		D0	D1	D2	D3	D4	D5	D6	D7	D14	D28	6 months
ENROLLMENT												
Eligibility screen	X											
Informed consent	X											
Randomization		X										
INTERVENTIONS												
Magnesium sulfate		X	X	X	X	X	X					
Placebo		X	X	X	X	X	X					
ASSESSMENTS												
ARDS	X	X	X	X	X	X	X	X	X			
Brain CT Imaging	X											
Magnesium level	X	X	X	X	X	X	X					
LAB												
ABG	X	X	X	X	X	X	X					
Blood tests	X											
Urine tests	X											
Biomarkers												
CRP	X				X				X			
GFAP	X				X				X			
IL-1β	X				X				X			
IL-6	X				X				X			
IL-8	X				X				X			
IL-10	X				X				X			
MDA		X			X				X			
sRAGE		X			X				X			
Substance P		X			X				X			
TNF-α		X			X				X			
UCH-L1		X			X				X			
GCS	X	X	X	X	X	X	X		X	X		
GOS-E											X	X
Survival status		X	X	X	X	X	X	X	X	X	X	X
Complications & Safety												
AEs/SAEs		X	X	X	X	X	X	X	X	X	X	
AKI		X	X	X	X	X	X	X	X	X	X	
Atrial fibrillation		X	X	X	X	X	X	X	X	X	X	
Hypermagnesemia		X	X	X	X	X	X					
Seizures		X	X	X	X	X	X	X	X	X	X	

ABG, Arterial Blood Gas Analysis; ARDS, Acute respiratory distress syndrome; AKI, Acute kidney injury; GCS, Glasgow Coma Scale; GOS-E, Glasgow Outcome Scale-Extended; SAE, Serious adverse event.

D0 is defined as within 8 h post-injury. The study intervention (magnesium sulfate or matching placebo) is administered once daily from D0 to D5 (total 6 doses). ARDS is assessed daily from D1 to D7 using the 2023 Delphi consensus criteria. Serum magnesium is monitored daily from D0 to D5; infusion is withheld if level >2.0 mmol/L (confirmed on two samples 4 h apart). GCS is assessed daily from D0 to D14. Biomarkers are collected at D0 (baseline, prior to intervention), D3, and D7. GOS-E is assessed at D28 and 6 months via blinded telephone interview. Complications (AKI, atrial fibrillation, seizures) are monitored daily during hospitalization and collected by telephone follow-up from discharge to D28.

**Figure 1:**
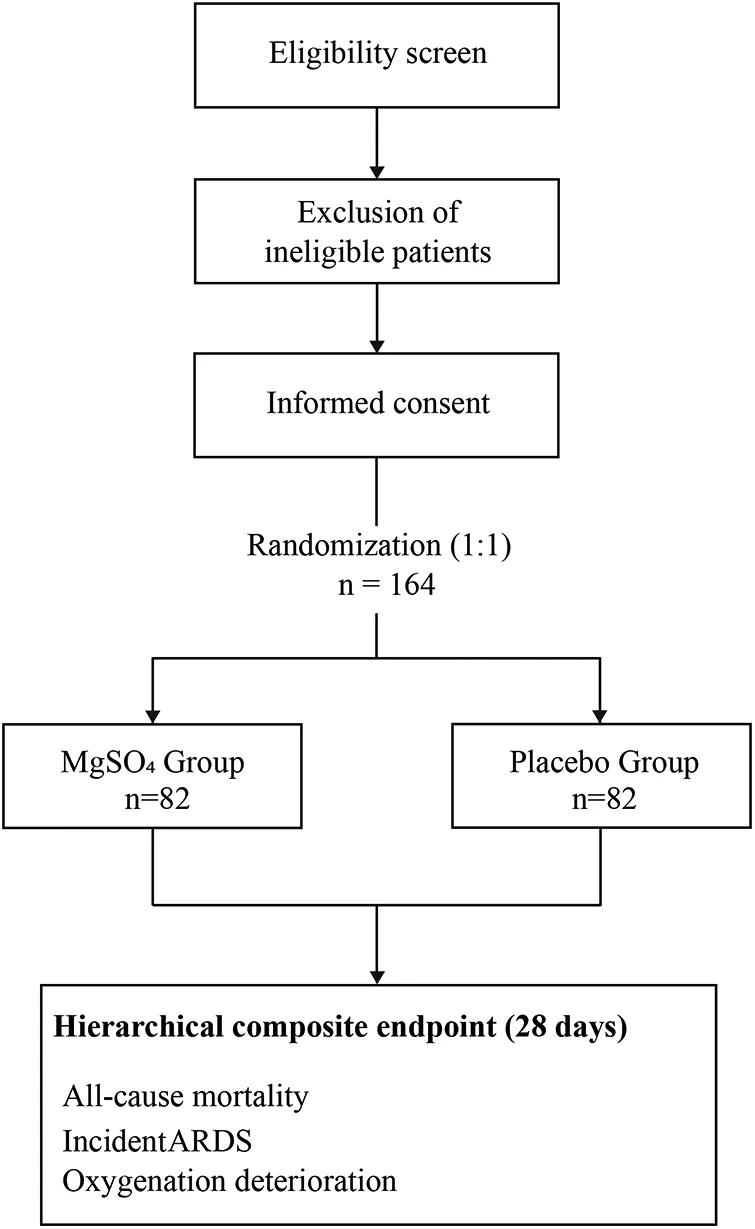
Overall study design and participant flow of the MOST trial. Eligible participants with moderate-to-severe traumatic brain injury (TBI) will undergo screening and informed consent before 1:1 randomization to receive either magnesium sulfate (MgSO₄) or placebo in addition to guideline-directed TBI care. The primary hierarchical composite endpoint will be assessed at 28 days and includes all-cause mortality, incident acute respiratory distress syndrome (ARDS), and oxygenation deterioration defined by the prespecified PaO₂/FiO₂ area-under-the-curve criterion.

**Figure 2:**
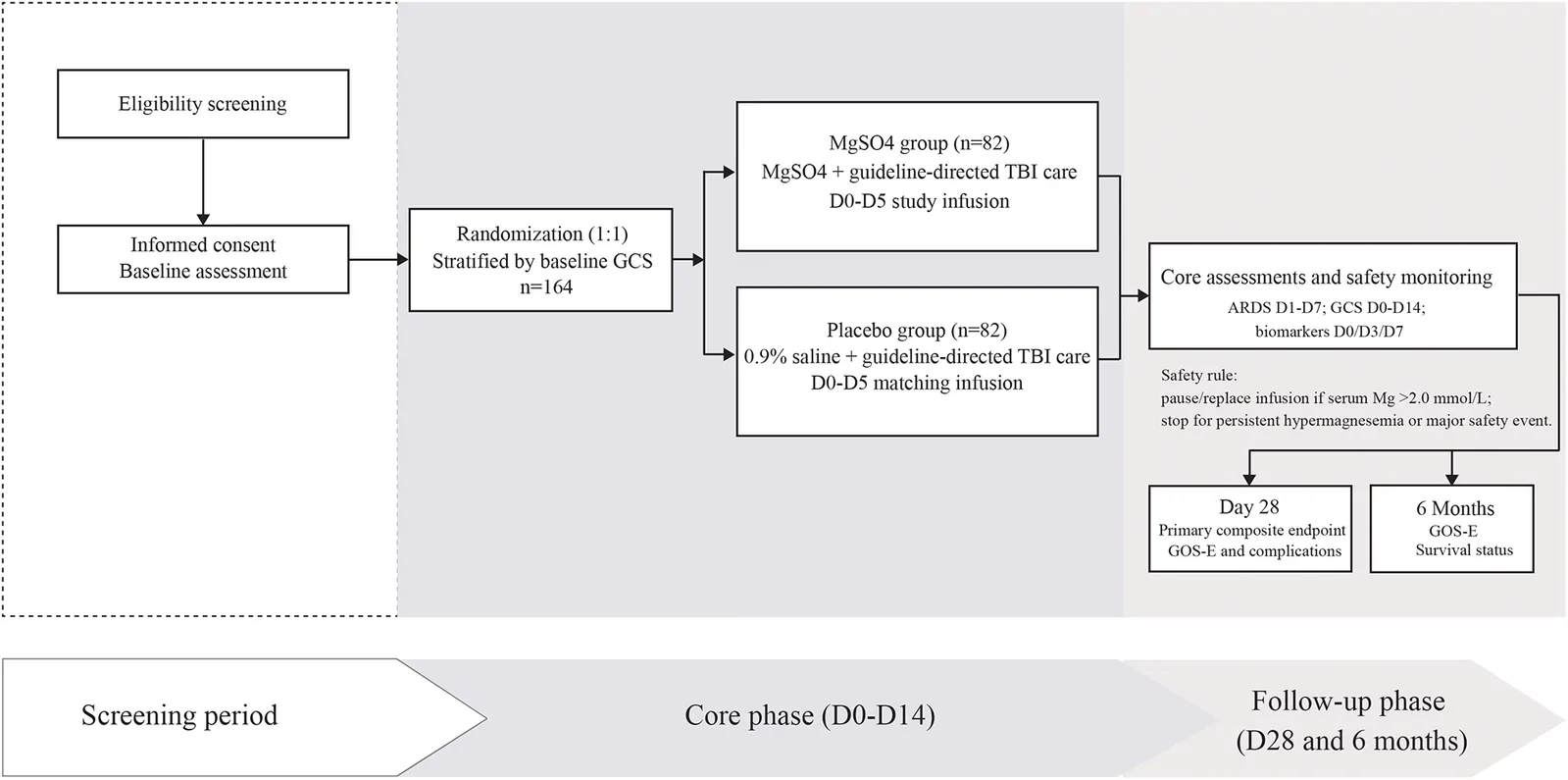
Detailed study timeline and assessment schedule of the MOST trial. The study consists of three periods: a screening period, a core treatment phase (day 0 to day 14), and a follow-up period extending to day 28 and 6 months after injury. After eligibility confirmation and baseline assessment, participants will undergo stratified randomization based on baseline Glasgow Coma Scale (GCS) score and receive MgSO₄ or placebo infusion in addition to guideline-directed TBI care. During the core treatment phase, serial clinical assessments, ARDS evaluation, safety monitoring, serum magnesium measurement, and biomarker assessments will be performed. The primary endpoint and short-term outcomes will be evaluated at day 28, and long-term neurological outcomes will be assessed at 6 months.

### Recruitment and eligibility

Participants will be recruited from the Emergency Department and the Neurocritical Care Unit (NCCU) of the study center, which is distinct from the general mixed intensive care unit (ICU).

#### Inclusion criteria

1. Age 18–75 years;
2. Moderate-to-severe traumatic brain injury (GCS 3–12 at admission) (29), confirmed by head CT or MRI showing intracranial injury (e.g., hematoma, cerebral oedema, or diffuse axonal injury);
3. Study intervention can be initiated within 8 h after injury (The time of injury will be determined from prehospital emergency medical service records, initial medical records from referring hospitals, or a clearly documented time provided by witnesses or family members. Patients for whom the time of injury cannot be reliably established or who present more than 8 h after injury will be excluded);
4. Hemodynamically stable, defined as systolic blood pressure ≥90 mmHg (vasopressor use permitted if ≤0.3 μg/kg/min norepinephrine equivalent);
5. Provision of written informed consent prior to study participation by the patient or their legally authorized representative

#### Exclusion criteria

1. Pre-existing acute respiratory distress syndrome (meeting 2023 Delphi consensus criteria) (30) or major aspiration/suspected aspiration pneumonia at screening;
2. Severe organ dysfunction or comorbidities: chronic kidney disease (eGFR <30 mL/min/1.73 m^2^), heart failure (NYHA class III-IV) (31), or decompensated cirrhosis (Child-Pugh class C) (32);
3. Known hypersensitivity or allergy to magnesium sulfate;
4. Baseline hypermagnesemia (serum magnesium concentration >1.5 mmol/L);
5. Refractory shock (requiring vasopressor >0.3 μg/kg/min norepinephrine equivalent with persistent hypotension and lactate >5 mmol/L), terminal brain herniation (midline shift >15 mm plus basal cistern obliteration on CT), or SOFA score ≥14;
6. Neurodegenerative diseases (e.g., Alzheimer's disease), uncontrolled epilepsy, myasthenia gravis, space-occupying brain tumor, or stroke within the previous year;
7. Severe concomitant trauma, including chest Abbreviated Injury Scale score ≥4 or lung contusion >20% of lung volume, emergency laparotomy for abdominal visceral rupture, flail chest, unstable spinal fracture requiring immediate surgery, or pelvic fracture with blood loss >1000 mL;
8. Pregnancy, lactation, or intention to conceive (in either sex) during the study period;
9. Participation in another interventional clinical trial within the past 30 days

Eligible participants will be recruited consecutively from the Emergency Department and Neurocritical Care Unit of the study center. All potential participants will undergo standardized screening against the predefined inclusion and exclusion criteria before enrollment. Enrollment criteria will be verified before randomization**,** and enrolled participants will be categorized according to baseline TBI severity as GCS 3–8 or GCS 9–12 for stratified randomization. The estimated recruitment period will be approximately 12 months.

### Randomization and grouping

Eligible participants will be randomly assigned in a 1:1 ratio to the intravenous magnesium sulfate (MgSO₄) group or the matching placebo (PBO) group using a stratified block randomization method, with baseline GCS score as the stratification factor (GCS 3–8 vs. GCS 9–12) and randomly varying block sizes of 4 to ensure balance and allocation concealment over time. Randomization will be conducted within 8 h after injury and before any surgical intervention.

The randomization sequence will be generated by an independent statistician using R with a prespecified random seed. The full R code used to generate the allocation sequence, together with the seed, will be archived to ensure reproducibility and auditability. The allocation list will be securely stored in an encrypted electronic file accessible only to the independent statistician. After eligibility confirmation and informed consent, the recruiting investigator will contact the statistician, who will assign the participant according to the predefined sequence and return the allocation result. Throughout the study, recruitment and outcome assessment personnel will remain blind to upcoming assignments. The allocation sequence will not be disclosed to any other study personnel, including investigators, outcome assessors, data managers, or statisticians not involved in sequence generation. Any modification of the randomization scheme is strictly prohibited.

### Blinding and emergency procedures

As a randomized, double-blind, placebo-controlled trial, participants, treating physicians, nursing staff, outcome assessors, and data analysts will remain blinded to treatment allocation throughout the study period.

Serum magnesium levels will be measured daily during treatment, with results transmitted directly from the laboratory to the unblinded pharmacist via an encrypted system. When serum magnesium exceeds the prespecified safety threshold (>2.0 mmol/L), the unblinded pharmacist will verify the result and prepare an equal-volume placebo using identical labeling and packaging. The blinded clinical team will remain unaware of the magnesium result and treatment substitution. The unblinded pharmacist will document the magnesium result and sampling/reporting time, threshold confirmation, study drug withholding/substitution, and placebo dispensing details in the unblinded pharmacy records. The blinded clinical team will document infusion time, administered volume, and treatment adherence in the eCRF without recording the magnesium result or substitution status. Pharmacy records and the eCRF will be reconciled after unblinding. If serum magnesium remains >2.0 mmol/L on a repeat measurement 4 h later, the investigator will also be notified, and the study intervention will be permanently discontinued for that participant in accordance with the protocol-defined stopping criteria.

Emergency unblinding will be permitted only in the event of a serious adverse event when knowledge of the assigned treatment is essential for clinical management. A sealed emergency unblinding card will be stored in the central pharmacy and opened in accordance with standard operating procedures. All unblinding events will be documented and reported to the Data and Safety Monitoring Board (DSMB) and the ethics committee. Final unblinding will be conducted after completion of the 28-day follow-up for all participants, database lock, and completion of blinded statistical analyses, and will be performed jointly by the DSMB chair and the independent statistician.

### Laboratory test

Laboratory tests include arterial blood gas analysis, routine blood and urine tests, and assays for inflammatory cytokines and biomarkers. Serum magnesium levels will be measured daily, with results sent directly from the laboratory to the unblinded pharmacist via an encrypted system; the pharmacist will not share these results with the study team, and if serum magnesium exceeds 2.0 mmol/L in a patient assigned to magnesium sulfate, the pharmacist will instruct the infusion to be paused and replaced with an equal volume of placebo for that day while the clinical team remains blinded, with no unblinding of the study team unless medically required. Serum magnesium in our protocol refers to total serum magnesium (33), measured daily via standard laboratory automated assays at our study center; ionized magnesium is not routinely available in our NCCU and was therefore not included in this protocol. Baseline and follow-up GCS scores will be assessed by two trained neurocritical care physicians who are independent of the randomization process and blinded to treatment allocation; in case of disagreement, a third blinded physician will adjudicate, and all GCS assessors will undergo pre-trial inter-rater reliability testing (target *κ* ≥ 0.80) and will not be involved in drug preparation or administration. Emergency unblinding may be performed only in the event of a serious adverse event where knowledge of the study drug is essential for patient management, and the unblinding request must be approved by the DSMB chair or ethics committee on-call representative and executed by the independent pharmacist, with any unblinding event documented and reported.

### Neuropsychological assessments

Neuropsychological assessments will be performed according to the standard timeline of the study (Table 1). The GCS will be assessed daily from Day 0 to Day 14 to monitor acute neurological status. The GCS score ranges from 3 to 15, with moderate-to-severe TBI defined as a score of 3 to 12 at enrollment (34). All GCS assessments will be conducted by two trained neurocritical care physicians, who are independent of the randomization process and blinded to treatment allocation. In case of disagreement, a third blind physician will adjudicate.

Long-term functional recovery will be evaluated using the Glasgow Outcome Scale-Extended (GOS-E) at two points: Day 28 and 6 months post-injury (Table 1). The GOS-E is an eight-point scale (ranging from 1 for death to 8 for upper good recovery) that assesses global functional outcome across seven domains, including consciousness, independence in daily living, work capacity, social and leisure activities, family relations, and return to normal life (35). GOS-E assessments will be performed by trained outcome assessors via blinded telephone interview or face-to-face follow-up, as appropriate.

All GCS and GOS-E assessors will undergo pre-trial training and inter-rater reliability testing (target *κ* ≥ 0.80) to ensure consistency. GOS-E scores will be dichotomized into favorable (scores 5–8) and unfavorable (scores 1–4) outcomes for secondary analysis.

### Interventions

Study drugs will be prepared by independent pharmacists at the central pharmacy according to the randomization assignment, using infusion bags and administration sets identical in appearance, labeling, and packaging. Labels will display only the study ID, participant ID, and infusion instructions. The infusion rate, frequency, and treatment duration will be identical in both groups.

Before surgical procedure, participants in both groups will receive standardized anesthesia and undergo neurosurgical intervention as clinically indicated. Anesthesia will be induced with intravenous propofol (1–2 mg/kg) or etomidate (0.2–0.3 mg/kg), selected at the clinician's discretion according to hemodynamic status, combined with remifentanil (0.5–1 μg/kg administered over 60 s) or sufentanil (0.2–0.5 μg/kg), and rocuronium (0.6–1.2 mg/kg) to facilitate tracheal intubation. Anesthesia will be maintained with a balanced regimen of intravenous propofol or inhaled sevoflurane (minimum alveolar concentration 0.8–1.2 MAC), supplemented with opioids and neuromuscular blocking agents as clinically required. Mechanical ventilation will be adjusted to maintain normocapnia (PaCO₂ 35–45 mmHg) before dural opening. Standard intraoperative monitoring will include electrocardiography, invasive arterial blood pressure, pulse oximetry, capnography, body temperature, and urine output. Neurosurgical procedures will be performed according to institutional standards and may include intracranial pressure monitor placement, decompressive craniectomy, or evacuation of intracranial hematoma, as determined by the attending neurosurgeon. Following surgical hemostasis and dural closure, anesthesia will be discontinued and the patient transferred to the intensive care unit for postoperative management. Serum magnesium levels will be monitored daily in all participants, with results available only to the unblinded pharmacist.

Both groups will receive identical guideline-directed standard care for moderate-to-severe traumatic brain injury, in accordance with the latest Brain Trauma Foundation guidelines (36), including intracranial pressure and cerebral perfusion pressure targets (ICP <22 mmHg and CPP 60–70 mmHg when monitored, with fallback MAP ≥80 mmHg), lung-protective ventilation, temperature management, and prophylaxis for seizures, stress ulcers, and venous thromboembolism.

Prohibited treatments include high-dose corticosteroids, and additional magnesium supplementation, except for protocol-defined rescue use in the PBO group. All concomitant medications will be recorded. Rescue magnesium supplementation is permitted in the placebo arm if serum magnesium falls below 0.75 mmol/L.

The first dose of the allocated study intervention will be initiated within 8 h of injury, prior to any surgical procedure, as described below.

#### MgSO₄ group

Participants randomized to the active arm will receive intravenous magnesium sulfate: an initial dose of 2 g in 100 mL 0.9% saline infused over 30 min (10 mL/h for the first 5 min, followed by 238 mL/h for the remaining 25 min if no infusion-related adverse reactions occur), followed by 2 g daily for 5 days (total 12 g). Infusions will be administered via a dedicated venous line using an infusion pump and must start within 8 h of injury. For patients requiring emergency neurosurgery, the first dose may be initiated before or during surgery, and study procedures will not delay any clinically indicated emergency surgery. Rescue magnesium supplementation is permitted only in the placebo arm if serum magnesium falls below 0.75 mmol/L, as determined by non-study clinicians. In the magnesium group, additional supplementation is not permitted unless serum magnesium decreases below 0.75 mmol/L and is considered clinically necessary, in which case it will be recorded as a protocol deviation. Serum magnesium levels will be monitored daily in all participants, with results available only to the unblinded pharmacist. The study infusion will be withheld if serum magnesium exceeds 2.0 mmol/L, interrupted for levels between 1.5 and 2.0 mmol/L and resumed once levels fall below 1.5 mmol/L, and permanently discontinued in the event of persistent hypermagnesemia, refractory hypotension, respiratory depression, anaphylaxis, participant withdrawal, or investigator safety concerns. Dose adjustment will be required only when eGFR is <30 mL/min/1.73 m^2^. Magnesium homeostasis will be monitored daily, with a target serum magnesium range of 1.0 to 1.5 mmol/L.

#### PBO group

The PBO group will receive an identical regimen of 100 mL 0.9% saline with the same schedule and rate.

#### Additional magnesium supplementation and monitoring

Rescue magnesium supplementation is permitted only in the PBO arm if serum magnesium falls below 0.75 mmol/L (decided by non-study clinicians). Although the frequency of rescue magnesium supplementation using this specific threshold has not been directly reported, a previous study of 156 patients with severe TBI found that 63.5% developed hypomagnesemia defined as serum magnesium <0.75 mmol/L, with a mean onset of 1.3 ± 0.4 days after injury (37). Based on these data, rescue magnesium supplementation could potentially be required in approximately 60%–65% of placebo-treated participants during the 5-day intervention period. In the magnesium arm, additional supplementation is prohibited unless serum magnesium drops below 0.75 mmol/L and the investigator deems it clinically necessary (documented as a protocol deviation). Serum magnesium levels are monitored daily in all participants, with results sent only to the unblinded pharmacist. The study infusion is withheld if serum magnesium exceeds 2.0 mmol/L (confirmed on two samples 4 h apart).

#### Criteria for discontinuing or modifying allocated intervention/comparator

The study infusion will be discontinued for serum magnesium >2.0 mmol/L (confirmed twice), refractory hypotension, respiratory depression, anaphylaxis, participant withdrawal, or investigator safety concern. It will be interrupted for levels 1.5–2.0 mmol/L and resumed when <1.5 mmol/L. Dose adjustment is required only if eGFR <30 mL/min/1.73 m^2^. Data will be retained for intention-to-treat analysis.

### Study endpoints

The primary outcome is a 28 day hierarchical composite, with components prioritized as all-cause mortality, followed by new onset ARDS within Days 1 to 7, defined according to the New Global Definition of ARDS (30), and oxygenation deterioration within Days 0 to 5 defined as a ≤ 20% increase from baseline in the area under the curve (AUC) of the ratio of partial pressure of arterial oxygen to fraction of inspired oxygen (PaO₂/FiO₂) (30, 38). This hierarchy was predefined according to clinical relevance and the expected progression spectrum of TBI-associated brain-lung injury. All-cause mortality was ranked first as the most severe and irreversible clinical outcome. New-onset ARDS was ranked second as a major pulmonary complication reflecting clinically significant brain-lung axis dysfunction. PaO₂/FiO₂ AUC change was ranked third as a continuous measure of oxygenation deterioration, allowing detection of pulmonary gas-exchange changes among pairs not distinguished at the preceding hierarchical levels. This hierarchical approach preserves the priority of clinically critical outcomes while incorporating informative physiological changes that may be overlooked by mortality-based assessment alone.

All suspected cases of ARDS will be reviewed by an independent endpoint adjudication committee consisting of two senior neurocritical care or critical care physicians who are blinded to treatment allocation. In cases of disagreement, a third independent expert will provide final adjudication. To distinguish ARDS from other causes of hypoxemia after severe TBI, including neurogenic pulmonary edema and cardiogenic pulmonary edema, adjudicators will consider the timing of pulmonary deterioration, clinical trajectory, response to therapy, and available cardiac assessments.

The secondary endpoints include the following: 1). Total duration (days) of ICU and hospital admission; 2). All-cause mortality within 28 days post-randomization; 3). Incidence of ECG-confirmed atrial fibrillation, clinically or EEG-diagnosed seizures, and Acute Kidney Injury (AKI) per KDIGO criteria (39); 4). Daily GCS scores from Day 0 to Day 14; 5). GOS-E scores (40) at 28 days and 6 months post-injury; 6). Adverse Events (AEs); 7). Mechanistic biomarkers, assessed by serial changes in circulating concentrations of predefined panels measured at Days 0, 3, and 7. The biomarker panel was prespecified to characterize biological processes potentially relevant to TBI-associated ARDS, including systemic inflammation, oxidative stress, neurogenic inflammatory signaling, and organ-specific injury responses: 1). Inflammatory Profile: interleukin-6 (IL-6), IL-8, tumor necrosis factor alpha (TNF-α), IL-1β, C-reactive protein (CRP), and IL-10; 2). Oxidative Stress & Neuropeptides: Malondialdehyde (MDA) and Substance P; 3). Brain Injury Markers: Glial fibrillary acidic protein (GFAP) and Ubiquitin carboxyl-terminal hydrolase L1 (UCH-L1); 4). Lung Epithelial Injury: Soluble receptor for advanced glycation end products (sRAGE). These biomarkers were chosen based on their established roles in neuroinflammation, oxidative stress, and brain-lung injury. The prespecified directional hypothesis was that, compared with placebo, MgSO₄ would attenuate post-treatment pro-inflammatory and oxidative stress responses, as reflected by lower levels of IL-6, IL-8, TNF-α, IL-1β, CRP, and MDA, with higher IL-10 levels reflecting a more favorable anti-inflammatory response; lower levels of GFAP, UCH-L1, sRAGE, and substance P were also expected, consistent with reduced neurological and pulmonary injury.

### Statistical analysis plan

All efficacy analyses will follow the intention-to-treat (ITT) principle, including all randomized participants according to their assigned treatment group regardless of protocol adherence. Missing data for the primary hierarchical endpoint will be handled according to the prespecified rules provided in Box 1. For secondary outcomes and covariates, multiple imputation will be used if the proportion of missing data is ≥5%; otherwise, complete-case analysis will be performed. Sensitivity analyses will be conducted to assess the robustness of the findings.

Box 1 **Hierarchical Comparison Rules for the Primary Outcome**

**Table:** 

**Hierarchical level**	**Comparison**	**Win/loss determination**	**Condition for proceeding to the next level**
Level 1: 28-day all-cause mortality	Compare survival status within 28 days; if both participants die, compare time to death.	If one participant survives to Day 28 and the other dies, the survivor wins. If both participants die, the participant with the longer survival time wins.	Proceed to Level 2 only if both participants survive to Day 28.
Level 2: New-onset ARDS during Days 1–7	Compare the occurrence of new-onset ARDS during Days 1–7.	If one participant develops ARDS and the other does not, the participant without ARDS wins.	If neither participant develops ARDS, or if both develop ARDS, this level is considered a tie and the comparison proceeds to Level 3.
Level 3: Oxygenation during Days 0–5	Compare the prespecified oxygenation measure based on PaO₂/FiO₂ AUC.	The participant with the more favorable oxygenation measure wins; identical values are considered a tie.	No further level. If the pair remains indistinguishable, the final result for that pair is a tie.
Calculation of PaO₂/FiO₂ AUC: Arterial blood gas analysis is performed once daily at six prespecified time points from Day 0 to Day 5. PaO₂/FiO₂ is calculated at each time point using the measured PaO₂ and the corresponding actual FiO₂. The Day 0–5 PaO₂/FiO₂ AUC is calculated using the linear trapezoidal method. For an isolated missing intermediate measurement, the PaO₂/FiO₂ value is imputed by linear interpolation between the adjacent available measurements. If the missing measurement occurs at Day 0 or Day 5, the nearest available value is used. If more than two measurements are missing, the AUC is considered unreliable and the participant is treated as a tie at Level 3. If PaO₂/FiO₂ measurements are unavailable because of palliative extubation or withdrawal of life-sustaining treatment, the participant is likewise treated as a tie at Level 3. An increase in AUC of ≤20% from the Day 0 baseline is defined as insufficient improvement or deterioration in oxygenation.			
Overall determination: Each participant pair is compared sequentially according to the prespecified hierarchy of 28-day all-cause mortality → new-onset ARDS during Days 1–7 → Day 0–5 PaO₂/FiO₂ AUC change, and the comparison stops at the first level at which a win or loss is determined. The final win ratio is calculated as the total number of pairs won by the MgSO₄ group divided by the total number of pairs lost by the MgSO₄ group.			

ARDS, acute respiratory distress syndrome; AUC, area under the curve; FiO₂, fraction of inspired oxygen; MgSO₄, magnesium sulfate; PaO₂, partial pressure of arterial oxygen.

Baseline characteristics will be summarized for all randomized participants according to the intention-to-treat population. Continuous variables will be presented as mean (standard deviation, SD) or median (interquartile range, IQR, 1st to 3rd quartile), as appropriate according to their distribution, and categorical variables will be presented as number (percentage). Baseline characteristics will be described by treatment groups to assess clinical comparability. In accordance with CONSORT recommendations (41), no formal statistical significance testing will be performed for between-group baseline comparisons.

The primary hierarchical composite endpoint will be analyzed using the Finkelstein-Schoenfeld win-ratio method (42, 43), with pairwise comparisons performed sequentially according to 28-day mortality status, occurrence of new ARDS, and oxygenation deterioration.

The Finkelstein-Schoenfeld method (42), operationalized as the win-ratio, is a statistical approach for analyzing hierarchical composite endpoints in clinical trials. It works by forming all possible pairs between treatment and control groups and comparing each pair sequentially according to a pre-specified hierarchy of outcomes, from most to least clinically important (e.g., first death, then new ARDS, then oxygenation deterioration). For each pair, a “win” is assigned to the group with the better outcome at the first level where a difference exists; ties proceed to the next level. The win-ratio is then calculated as the total number of pairs in which the treatment group wins divided by the total number of pairs in which the control group wins, with a win-ratio >1 indicating treatment benefit. This method preserves the clinical priority of outcomes, avoids the equal-weighting problem of traditional composite endpoints, and provides an intuitive interpretation. Detailed rules for the hierarchical comparison of the primary outcome are provided in Box 1.

Secondary outcomes will be analyzed according to endpoint type. Continuous outcomes, including ICU and hospital length of stay, GOS-E scores, serum magnesium concentrations, and circulating biomarker levels, will be analyzed using either Student's t-test or the Mann–Whitney U test, as appropriate based on data distribution. Repeated-measures outcomes, including daily GCS scores (Days 0–14) and serial biomarker measurements (Days 0, 3, and 7), will be analyzed using linear mixed-effects models with fixed effects for treatment group, time, and group-by-time interaction. Binary and categorical outcomes, including 28-day mortality, atrial fibrillation, seizures, acute kidney injury, adverse events, and serious adverse events, will be compared using the chi-squared test or Fisher's exact test, as appropriate. Safety outcomes, including hypermagnesemia, hypotension, respiratory depression, loss of deep tendon reflexes, atrial fibrillation, seizures, and acute kidney injury, will be summarized descriptively and compared between groups using appropriate statistical methods. The prespecified adverse events of special interest (AESIs) and their severity grading criteria are summarized in Box 2. All secondary endpoint analyses will be considered exploratory, and corresponding *p* values will be reported as nominal only and will not be used for formal statistical inference.

Box 2 **Prespecified Adverse Events of Special Interest and Severity Grading Criteria**

**Table:** 

**AESI**	**Grade**	**Grading criteria**
Hypermagnesemia: Serum magnesium above ULN	Grade 1: >ULN to 1.5 mmol/L; Grade 2: >1.5 to 2.0 mmol/L; Grade 3: >2.0 to 3.0 mmol/L; Grade 4: >3.0 mmol/L; Grade 5: Death related to AE	protocol-defined
Hypotension: SBP <90 mmHg or MAP <65 mmHg	Grade 1: Asymptomatic, no intervention required; Grade 2: Symptomatic, responds to IV fluid bolus; Grade 3: Requires vasopressor support; Grade 4: Life-threatening shock requiring escalation of vasopressors; Grade 5: Death related to AE	NCI CTCAE v5.0
Respiratory depression: RR <10/min or SpO₂ < 90% on room air	Grade 1: Asymptomatic or mild; Grade 2: Symptomatic, requires low-flow supplemental oxygen; Grade 3: Requires non-invasive positive-pressure ventilation (e.g., BiPAP); Grade 4: Life-threatening, requires immediate endotracheal intubation and mechanical ventilation; Grade 5: Death related to AE	NCI CTCAE v5.0
Loss of deep-tendon reflexes: Patellar/biceps reflex abnormality	Grade 1: Hyporeflexia (1+); Grade 2: Complete loss of reflex (0) without motor deficit; Grade 3: Loss of reflex accompanied by motor weakness (muscle strength <=3/5); Grade 4: Loss of reflex accompanied by acute flaccid paralysis or respiratory muscle involvement; Grade 5: Death related to AE	protocol-defined
Bradycardia/conduction block: Bradycardia or AV conduction abnormality	Grade 1: asymptomatic, HR 45–50 bpm or PR prolongation on ECG; Grade 2: symptomatic, HR <45 bpm without hemodynamic collapse; Grade 3: severe AV block or HR <40 bpm requiring temporary pacing; Grade 4: life-threatening arrhythmia requiring urgent intervention; Grade 5: Death related to AE	protocol-defined
Atrial fibrillation:
ECG-confirmed or clinically documented new-onset or recurrent atrial fibrillation	Grade 1: Asymptomatic, no intervention required; Grade 2: Symptomatic, non-urgent medical intervention required (e.g., rate control, anticoagulation); Grade 3: Incomplete hemodynamic stability, urgent medical intervention required; Grade 4: Life-threatening consequences, urgent electrical cardioversion or hemodynamic support required; Grade 5: Death related to AE	NCI CTCAE v5.0
Seizures:
Clinical or electroencephalographic (EEG) evidence of new-onset or recurrent seizure activity	Grade 1: Brief or isolated seizure, self-limiting, no anti-seizure medication required; Grade 2: Brief seizure(s) controlled with a single anti-seizure medication; Grade 3: Recurrent or prolonged seizures requiring multiple anti-seizure medications or intravenous therapy; Grade 4: Life-threatening status epilepticus requiring endotracheal intubation or ICU admission; Grade 5: Death related to AE	NCI CTCAE v5.0
Acute kidney injury:
Acute increase in serum creatinine or reduction in urine output	Grade 1: Serum creatinine >0.3 mg/dL (>26.5 μmol/L) or 1.5–2.0 × baseline; Grade 2: Serum creatinine >2.0–3.0 × baseline; Grade 3: Serum creatinine >3.0 × baseline or >4.0 mg/dL (>354 μmol/L), hospitalization indicated; Grade 4: Life-threatening consequences, dialysis or renal replacement therapy indicated; Grade 5: Death related to AE	NCI CTCAE v5.0


AESI, adverse event of special interest; AV, atrioventricular; CTCAE, Common Terminology Criteria for Adverse Events; ECG, electrocardiogram; EEG, electroencephalography; HR, heart rate; MAP, mean arterial pressure; NCI, National Cancer Institute; PR, PR interval; RR, respiratory rate; RRT, renal replacement therapy; SBP, systolic blood pressure; SpO2, peripheral oxygen saturation; ULN, upper limit of normal. Severity grading was based on NCI CTCAE v5.0 where applicable, supplemented by protocol-defined criteria for events without a directly applicable CTCAE category.

The primary endpoint analysis will be performed using the Intention-to-Treat (ITT) set as the primary analysis population, with all randomized participants analyzed according to their assigned treatment group. Missing data for the primary endpoint will be conservatively imputed as the worst outcome. Missing covariate data will be addressed using multiple imputation methods. The Full Analysis Set (FAS) will be used as a sensitivity analysis population to evaluate the robustness of the primary findings.

Any emergency unblinding required for safety reasons will be fully documented and reported to the DSMB and ethics committee according to predefined procedures. Such events will not result in exclusion from the primary analysis and will be analyzed according to the ITT principle.

### Sample size

The sample size assumptions were derived from a retrospective analysis of the MIMIC-IV database, which evaluated differences in mortality, ARDS incidence, and oxygenation trajectories according to serum magnesium levels (44), combined with published literature on TBI-associated ARDS incidence (6). Based on the preliminary hierarchical pairwise comparison incorporating 28-day mortality, new-onset ARDS, and PaO₂/FiO₂ AUC, the anticipated cumulative win proportions were 58% for the MgSO₄ group and 35% for the placebo group, with approximately 7% of pairwise comparisons remaining tied after all three hierarchical levels. Accordingly, the anticipated win ratio was 0.58/0.35 ≈ 1.66.

For sample size estimation, the anticipated cumulative win proportions of 0.58 and 0.35 were used, with a two-sided *α*=0.05, 80% power, and a 1:1 allocation ratio. The calculation yielded a required sample size of 73 participants per group (146 participants in total). After allowing for an anticipated 10% loss to follow-up or nonadherence, the target sample size was increased to 82 participants per group, resulting in a total planned enrollment of 164 participants.

### Interim analyses

No formal interim efficacy analysis is planned. The independent DSMB will conduct safety reviews after approximately 25%, 50%, and 75% of the planned sample has completed the 28-day follow-up. The DSMB will review accumulating mortality, serious adverse events, and other relevant safety data and may recommend temporary suspension or early termination of the trial if a clinically important safety concern or unfavorable safety imbalance between treatment groups is identified. Any such recommendation will be promptly submitted to the Ethics Committee for review, and the final decision regarding temporary suspension or early termination of the trial will be made by the Ethics Committee.

### Data management, quality control, and study adherence

All participant data will be entered and managed in the ResMan EDC system, which provides built-in validation, role-based access control, daily encrypted backups, and a complete audit trail. Data entry will follow predefined coding rules with automated range and consistency checks. Regular monitoring will include source data verification, discrepancy resolution, and protocol compliance review. Study adherence and protocol fidelity will be supported through standardized staff training, daily electronic checklists within the ResMan system, timely pharmacy preparation of study infusions, twice-weekly quality audits, and monthly investigator review meetings. All data will be stored in accordance with institutional policies and applicable data protection regulations. The finalized protocol and a concise statistical analysis plan have been posted on the Chinese Clinical Trial Registry.

### Ethics and dissemination

The final protocol version 3.0 for this study has been approved by the Ethics Committee of Shanxi Bethune Hospital (approval number: LYLL-2026-00L/PJ030, dated 2 March 2026). The trial will be conducted in strict accordance with the Declaration of Helsinki and the International Council for Harmonization Good Clinical Practice (ICH-GCP) guidelines. Written informed consent will be obtained from all participants, or from their legally authorized representative if the participants lack capacity for enrolment, with re-consent sought once capacity is regained. All study procedures are applied uniformly, irrespective of sex, race, or age.

The trial has been registered with the Chinese Clinical Trial Registry (ChiCTR2600120684) and in the National Medical Research Registration and Record Information System of China (MR-14-26-018983). Trial results will be disseminated transparently regardless of positive, neutral, or negative findings. Findings will be submitted for publication in peer-reviewed journals and updated in the primary clinical trial registry.

## Discussion

The pathophysiology of TBI associated ARDS involves sympathetic surge, systemic inflammation, oxidative stress, and the neural-respiratory inflammasome axis (45, 46). Magnesium sulfate could theoretically modulate these processes through effects on calcium homeostasis, endothelial integrity, and anti-inflammatory pathways, thereby potentially influencing the brain-lung interactions underlying TBI-associated ARDS (18, 47, 48).

However, clinical evidence in TBI remains inconclusive, with prior trials focusing on neurological rather than pulmonary outcomes and showing mixed results (20, 49–51). Unlike previous studies, in which the primary outcomes included cerebral oxygen saturation, GOS, or mortality risk (52–54) the MOST trial employs a hierarchical composite primary endpoint comprising all-cause mortality, new-onset ARDS, and change in PaO₂/FiO₂ AUC. This endpoint design allows the therapeutic effects of magnesium sulfate in TBI to be reevaluated from a different clinical perspective, with particular emphasis on pulmonary complications and oxygenation deterioration. The dose and target serum magnesium concentration in the MOST trial were selected with consideration of both the distinct therapeutic objective and the safety findings of previous TBI trials. Temkin et al. evaluated substantially higher serum magnesium targets for neuroprotection but found no improvement in neurological outcomes, while higher magnesium exposure was associated with safety concerns (20). Given that MOST focuses on TBI-associated pulmonary injury and brain-lung axis rather than neuroprotection, we did not seek to reproduce these supraphysiological magnesium targets. Instead, the target serum magnesium concentration of 1.0–1.5 mmol/L represents a conservative exposure strategy intended to provide potential modulation of inflammation, oxidative stress, and endothelial and epithelial injury while limiting the risks of hypermagnesemia, hypotension, and respiratory depression. However, this regimen remains exploratory, and potential under-dosing cannot be excluded. The present phase II trial will therefore also provide safety and mechanistic data to inform dose selection in future studies.

The MOST trial is the first dedicated investigation of early intravenous magnesium sulfate administration aimed at interrupting brain-lung axis and preventing ARDS in patients with moderate-to-severe TBI. Its major strengths include the utilization of a clinically meaningful hierarchical composite endpoint analyzed using the win-ratio method, the incorporation of serial mechanistic biomarkers, rigorous double-blinding, and the evaluation of a low-cost, easily implementable intervention that can be readily integrated into existing TBI care pathways. If effective, this trial would support early prophylactic magnesium sulfate, paving the way for larger confirmatory trials and potential guideline updates. Conversely, a negative result would still provide mechanistic insights and inform dosing or delivery strategies in future studies.

Limitations of the study include the single-center design and modest sample size (*n* = 164), which may limit generalizability. These limitations are partly mitigated by 6-month functional follow-up, rigorous procedures, and planned multi-center extension. Exploratory analyses of additional pathways (e.g., HMGB1, TRPM7) using banked biospecimens will be pursued in subsequent studies. Additionally, while total serum magnesium may not fully reflect biologically active ionized magnesium in critically ill patients (33), ionized magnesium was not routinely available in our clinical setting. Future studies incorporating ionized magnesium measurements may further clarify the relationship between magnesium status and clinical outcomes. Regardless of the primary results, the MOST trial will generate high-quality data on magnesium-based therapy and the brain-lung axis in acute brain injury.

## Conclusion

The MOST trial is designed to evaluate whether early intravenous magnesium sulfate can reduce the risk of ARDS and improve clinical outcomes in patients with moderate-to-severe traumatic brain injury. By integrating a hierarchical composite endpoint, mechanistic biomarker profiling, and rigorous double-blind methodology, this study may provide new evidence regarding the role of magnesium sulfate in modulating the brain-lung axis after acute brain injury. If successful, this low-cost and widely accessible intervention could be readily incorporated into standard neurocritical care practice and inform future multicenter confirmatory trials and guideline development.
